# Tobacco smoke exposure and sleep problems: A secondary dataset analysis of NHANES and Mendelian randomization analyses

**DOI:** 10.18332/tid/220984

**Published:** 2026-08-01

**Authors:** Xindong Zhu, Chunming Jiang

**Affiliations:** 1 The Fourth school of Clinical Medicine, Zhejiang Chinese Medical University, Hangzhou First People’s Hospital, Hangzhou, China; 2 Department of Pediatrics, Affiliated Hangzhou First People’s Hospital, School of Medicine, Westlake University, Hangzhou, China

**Keywords:** NHANES, tobacco smoke exposure, sleep deprivation, sleep disorder, Mendelian randomization

## Abstract

**INTRODUCTION:**

Smoking and sleep problems are both major public health concerns, but the causal nature of their association remains unclear due to confounding and reverse causality. To address this, observational and Mendelian randomization approaches were combined to investigate the causal relationships between smoking status, cotinine levels, and sleep deprivation and sleep disorder.

**METHODS:**

Data from NHANES (2005–2020) were analyzed using weighted logistic regression, subgroup, and threshold analyses to assess associations between smoking status, cotinine levels, and sleep deprivation and sleep disorder. Mendelian randomization was then performed using genetic instruments for smoking phenotypes to evaluate causal relationships.

**RESULTS:**

In NHANES (n=22380), current smokers showed significantly higher risk of sleep disorder (OR=1.68; 95% CI: 1.49–1.90) and sleep deprivation (OR=1.52; 95% CI: 1.36–1.70) compared to never smokers. Serum cotinine was positively associated with both outcomes in a nonlinear manner, with threshold effects observed. In Mendelian randomization, genetic predisposition to current smoking was causally associated with increased risk of sleep deprivation (OR=1.253; 95% CI: 1.015–1.548), while never smoking showed protective causal effects on both outcomes. However, no causal associations were found between cotinine and sleep outcomes.

**CONCLUSIONS:**

Observational analyses revealed associations between smoking status, serum cotinine levels, and both insufficient sleep and sleep disorders. However, while Mendelian randomization provided evidence supporting a causal relationship between smoking status and sleep outcomes, it did not establish a causal link between serum cotinine and either insufficient sleep or sleep disorders.

## INTRODUCTION

Sleep disorders comprise a range of conditions marked by disruptions in sleep quality, timing, or duration, frequently leading to daytime impairment and reduced well-being. Based on the American Academy of Sleep Medicine (AASM) classification, these disorders are grouped into six primary categories: insomnia, sleep-related breathing disorders, central disorders of hypersomnolence, circadian rhythm sleep–wake disorders, parasomnias, and sleep-related movement disorders^1^. As a prevalent public health concern, sleep disorders considerably diminish quality of life and overall health. A cohort study of 284754 US adults followed for up to ten years indicated that sufficient sleep duration offers protective health benefits, whereas short sleep is associated with elevated all-cause mortality^2^. Accumulating evidence also suggests that sleep disorders are strongly associated with an increased risk of numerous adverse outcomes, such as cardiovascular diseases (such as hypertension, myocardial infarction, stroke), metabolic disorders (such as type 2 diabetes and obesity), immune dysfunction, and neuropsychiatric illnesses (e.g. depression and anxiety)^3,4^. Furthermore, inadequate sleep adversely affects cognitive function, attention, and memory, raises the incidence of accidents, and substantially impairs both quality of life and work performance, contributing to significant healthcare burdens and socioeconomic costs.

Among modifiable risk factors for sleep disorders, tobacco use remains a leading preventable cause of disease worldwide^5^. While the adverse effects of smoking on respiratory and cardiovascular health are well-established, its impact on sleep has recently attracted growing research interest^6^. Accumulating evidence suggests that tobacco exposure – whether through active smoking, secondhand, or thirdhand smoke – may contribute to sleep disturbances^7^. Several epidemiological studies have further demonstrated a significant correlation between higher levels of tobacco exposure and reduced sleep duration^8,9^, suggesting a possible relationship. Moreover, a systematic review indicated that smokers show a decreased proportion of deep sleep (slow-wave sleep) and disrupted sleep continuity, as evidenced by electroencephalographic findings^10^.

However, existing studies exhibit notable limitations. A primary issue is their reliance on self-reported smoking behavior, which is subject to substantial underreporting bias as confirmed in behavioral research, resulting in systematic underestimation of actual tobacco exposure by approximately 30–50%^11^. Moreover, conventional questionnaires frequently fail to account for non-smokers’ exposure to secondhand smoke. In contrast, cotinine – the principal metabolite of nicotine, representing approximately 70–80% of its metabolic products^12,13^ – is a reliable and widely used biomarker of tobacco exposure for objectively quantifying internal tobacco exposure due to its relatively long half-life (approximately 16–20 hours) and stable metabolic characteristics.

Currently, research investigating the association between sleep disorders and cotinine as an objective measure of tobacco exposure, remains limited. Therefore, building upon preliminary analyses of self-reported smoking behavior in relation to sleep disorders and sleep duration, this study utilizes serum cotinine levels as a core exposure variable to evaluate this association more accurately and objectively. Furthermore, to mitigate confounding biases and reverse causality inherent in observational designs, we employ Mendelian randomization (MR), which uses genetic variants – primarily single-nucleotide polymorphisms (SNPs) – that are strongly associated with the exposure as instrumental variables (IVs).

Against this background, the present study first assesses the relationship between smoking behavior and sleep disorders using NHANES data, and further examines the association of serum cotinine levels with sleep disorders along with its potential causal nature. Specifically, observational analyses based on cross-sectional data from the NHANES were performed to assess phenotypic associations between smoking behavior, serum cotinine, and sleep disorders. Subsequently, this study applied a two-sample MR framework to investigate the effects of genetically predicted smoking behavior and serum cotinine levels on sleep disorders.

## METHODS

This study employed a dual analytic approach. First, a secondary data analysis was conducted using the cross-sectional NHANES dataset (2005–2020) to assess associations between smoking status, serum cotinine levels, and sleep outcomes. Second, a two-sample MR study was performed using genetic instruments for smoking phenotypes and cotinine levels from the EBI database to evaluate relationships. Detailed descriptions of each method are provided below.

### Secondary dataset analysis

*Data selection and study design*

The data for this study were derived from the NHANES, a nationally representative cross-sectional survey program conducted by the Centers for Disease Control and Prevention (CDC) to systematically monitor the health and nutritional status of adults and children in the United States. NHANES employs a complex, stratified, multistage probability sampling design to ensure national representativeness.

Data from seven two-year cycles of NHANES between 2005 and 2020 were included. The inclusion criteria were: 1) complete questionnaire data on smoking status and serum cotinine measurements; 2) clearly documented sleep disorder diagnoses and sleep duration records; and 3) availability of all covariate data. Exclusion criteria were: 1) missing smoking status or serum cotinine data; 2) incomplete sleep disorder diagnosis or sleep duration information; 3) missing key covariates; and 4) diagnosis of cancer or current pregnancy. We finally included 22380 participants in the present analysis (Supplementary file Figure 1).

*Exposure: Assessment of smoking status and serum cotinine*

In the NHANES study, smoking status was determined based on two interview questions: ‘Have you smoked at least 100 cigarettes in your entire life?’ and ‘Do you now smoke cigarettes?’. Based on participants’ responses, smoking status was categorized into three groups: never smokers (smoked fewer than 100 cigarettes in their lifetime), former smokers (smoked at least 100 cigarettes but do not currently smoke), and current smokers (smoked at least 100 cigarettes and currently smoke). Serum cotinine levels were measured using high-precision isotope dilution high-performance liquid chromatography/atmospheric pressure chemical ionization tandem mass spectrometry (ID HPLC-APCI-MS/MS). This method incorporates stable isotope-labeled internal standards for quantitative correction and employs high-performance liquid chromatography for effective separation of metabolites, significantly enhancing the accuracy and reliability of the detection.

*Outcome: Assessment of sleep disorder and sleep inefficiency*

In this study, sleep disorders were assessed using the relevant survey question from NHANES: ‘Have you ever been told by a doctor or other health professional that you have a sleep disorder?’. Participants who responded ‘Yes’ were classified as having a sleep disorder, while those who answered ‘No’ were classified as without a sleep disorder. Responses such as ‘Refused’, ‘Don’t know’, or missing data were treated as missing values and excluded from the analysis. Sleep duration was assessed using the question, ‘How much sleep do you usually get at night on weekdays or workdays?’. Based on recommendations from the American Academy of Sleep Medicine (AASM), participants reporting less than 7 hours of sleep per night were defined as having insufficient sleep^14^. All questions were administered by trained interviewers using the Computer-Assisted Personal Interviewing (CAPI) system in participants’ homes. The CAPI system incorporates built-in consistency checks to help reduce data entry errors. This sleep disorder questionnaire has been widely used in multiple studies and has demonstrated good reliability and validity^15,16^.

*Covariates*

Based on a comprehensive literature review^17^, this study selected multi-dimensional variables that may influence sleep disorders and sleep duration in the population as covariates, covering two major aspects: demographic characteristics and physical health conditions. Among the demographic variables, the following covariates were included: age, sex, race/ethnicity (Mexican American, Other Hispanic, Non-Hispanic White, Non-Hispanic Black, and Other), family income-to-poverty ratio (PIR; with PIR ≥2 considered as non-poor and PIR <2 as poor), education level, and marital status. In terms of physical health variables, self-reported presence of underlying diseases such as hypertension, heart disease, diabetes, stroke, and asthma – obtained through standardized questionnaires – was included. Additionally, physical activity level (PA: <600, 600–1200, and >1200 MET-min/week), daily dietary intake (daily energy intake was estimated from 24-hour dietary recalls using the USDA Food and Nutrient Database for Dietary Studies (FNDDS) to obtain standardized nutrient values and calculate total calories), and body mass index (BMI, kg/m^2^) , were incorporated as covariates.

*Statistical analysis*

All statistical analyses incorporated sampling weights provided by NHANES to ensure representativeness of the non-institutionalized US population. Data processing and analysis were performed using R 4.2.1, with a two-sided significance level of α=0.05. In accordance with the NHANES official analytical guidelines, sampling weights, stratification, and clustering variables were incorporated in all analyses to account for the complex multistage sampling design^18^. Continuous variables are presented as mean ± standard deviation (SD), and categorical variables as frequencies with weighted percentages. Group comparisons were conducted using weighted linear regression (for continuous outcomes) or weighted multivariable logistic regression (for categorical outcomes), as appropriate. All hypothesis tests were two-sided, and a p<0.05 was considered statistically significant.

First, smoking status was included as the exposure variable, categorized into three groups (never smokers, former smokers, and current smokers), with the presence of sleep disorders and insufficient sleep as outcomes. Weighted multivariable logistic regression models were applied to evaluate these associations

Subsequently, weighted multivariable logistic regression was also used to examine the associations between serum cotinine levels and the risk of sleep disorders and insufficient sleep. To explore potential heterogeneity, subgroup analyses were conducted by gender, race/ethnicity, age, education level, BMI category, and other factors. Threshold effect analysis was performed to identify potential nonlinear relationships, with inflection points determined and piecewise logistic regression models applied to assess threshold effects. Serum cotinine was also divided into tertiles (No tobacco exposure: serum cotinine <0.05 ng/mL; Low tobacco exposure: serum cotinine: 0.05–3 ng/mL; High tobacco exposure: serum cotinine >3 ng/mL) and included in the models as a categorical variable for validation. Three models were constructed: Crude Model was unadjusted; Model 1 adjusted for age, gender, educational level, PIR, and marital status; and Model 2 further adjusted for daily dietary energy intake, self-reported general health status (including chronic conditions such as hypertension, heart disease, diabetes, and stroke), physical activity level, and alcohol consumption.

### Mendelian randomization study

*Overview*

MR analysis relies on three core assumptions for causal inference: 1) the instrumental variables (IVs) must be strongly associated with the exposure; 2) the IVs should be independent of potential confounders; and 3) the IVs should influence the outcome only through the exposure^19^. It is noteworthy that all genome-wide association studies (GWAS) included in the analysis had received approval from their respective ethics review boards; therefore, no additional ethical approval or participant consent was required

*Genetically instrumental variables for smoking status and serum cotinine in MR*

This study utilized publicly available large-scale GWAS summary statistics. Genetic instruments for smoking behaviors (never smoking, former smoking, and current smoking) were obtained from the IEU database. Genetic instruments for serum cotinine were sourced from the EBI database, which included data from 5185 individuals of European ancestry across various age groups. The following standard procedures were applied to select SNPs for each exposure, ensuring compliance with the core assumptions of MR analysis. First, SNPs significantly associated with the exposures were selected using a threshold of p<5×10^-8^ for smoking behaviors and p<5×10^-6^ for serum cotinine. Next, SNPs in linkage disequilibrium (LD) were pruned using parameters r^2^<0.001 and a clumping distance of 10000 kb to ensure independence among instruments. Finally, the F-statistic was calculated for each SNP, with F >10 considered indicative of a strong instrument and a robust association with the exposure. The overall workflow of the MR analysis is illustrated in Supplementary file Figure 2.

### Genetic summary data on sleep disorder in MR

Summary statistics for GWAS related to sleep disorders and insufficient sleep were obtained from the IEU database. The GWAS dataset for sleep disorders included 2915 individuals of European ancestry, covering all age groups and both sexes. The GWAS dataset for insufficient sleep comprised 28980 individuals, also of European descent and including both males and females.

### Statistical analyses

The effects of smoking status and serum cotinine levels on sleep disorders and their subtypes were primarily evaluated using the inverse-variance weighted (IVW) method. To verify the robustness of the IVW results, supplementary analyses were conducted with MR-Egger, weighted median, weighted mode, and simple mode methods. Sensitivity analyses were performed using IVW and MR-Egger to assess heterogeneity among instrumental variables (IVs), with the extent of heterogeneity quantified by the p-value of Cochran’s Q test; a p<0.05 indicated heterogeneity. It should be noted that the presence of heterogeneity does not necessarily invalidate the IVW results. To evaluate potential horizontal pleiotropy, MR-Egger regression and MR-PRESSO global tests were applied to examine the intercept term; results showed no evidence of horizontal pleiotropy (p>0.05). Additionally, a leave-one-out analysis was performed to identify any single SNP exerting an outsized influence on the IVW estimates. Finally, funnel plot symmetry was visually inspected to assess the reliability of the results. All analyses were carried out using the TwoSampleMR and MR-PRESSO packages in R software (version 4.2.1).

## RESULTS

### NHANES observational study

*Baseline characteristics of the study participants in NHANES*

Table 1 presents the characteristics of participants from NHANES 2005–2020 by smoking status. Participants were categorized into three groups based on smoking status: non-smokers (12277; 54.9%), former smokers (5548; 24.8%), and current smokers (4555; 20.3%). Analysis of baseline characteristics revealed that former smokers had a significantly higher mean age (57.23 ± 16.61 years) compared to

**Table 1:** Baseline characteristics of participants, by smoking status, NHANES 2005–2020 (N=22380)

*Characteristics*	*Total* *(N=22380)* *n (%)*	*Non-smoker* *(N=12277)* *n (%)*	*Former smoker* *(N=5548)* *n (%)*	*Current smoker* *(N=4555)* *n (%)*	*p*
**Age** (years), mean ± SD	49.06 ± 17.64	47.13 ± 17.75	57.23 ± 16.61	44.31 ± 15.16	<0.001
**Gender**					<0.001
Male	12,155 (54.3)	6,505 (53.0)	3,243 (58.5)	2,407 (52.8)	
Female	10225 (45.7)	5772 (47.0)	2305 (41.5)	2148 (47.2)	
**Race**					<0.001
Mexican American	3363 (15.0)	2105 (17.1)	755 (13.6)	503 (11.0)	
Non-Hispanic Black	4527 (20.2)	2580 (21.0)	850 (15.3)	1097 (24.1)	
Non-Hispanic White	10417 (46.5)	4998 (40.7)	3110 (56.1)	2309 (50.7)	
Other Hispanic	2077 (9.3)	1255 (10.2)	489 (8.8)	333 (7.3)	
Other	1996 (8.9)	1339 (10.9)	344 (6.2)	313 (6.9)	
**Education level**					<0.001
High school	5040 (22.5)	2435 (19.8)	1281 (23.1)	1324 (29.1)	
Lower than high school	5117 (22.9)	2516 (20.5)	1249 (22.5)	1352 (29.7)	
Some college or higher	12223 (54.6)	7326 (59.7)	3018 (54.4)	1879 (41.3)	
**Marital status**					<0.001
Married/living with partner	14401 (64.3)	8161 (66.5)	3706 (66.8)	2534 (55.6)	
Never married	2996 (13.4)	1747 (14.2)	484 (8.7)	765 (16.8)	
Widowed/divorced/separated	4983 (22.3)	2369 (19.3)	1358 (24.5)	1256 (27.6)	
**PIR**					<0.001
<2	10397 (46.5)	5217 (42.5)	2317 (41.8)	2863 (62.9)	
≥2	11983 (53.5)	7060 (57.5)	3231 (58.2)	1692 (37.1)	
**Angina pectoris**					<0.001
Yes	548 (2.4)	212 (1.7)	210 (3.8)	126 (2.8)	
No	21832 (97.6)	12065 (98.3)	5338 (96.2)	4429 (97.2)	
**Coronary artery disease**					<0.001
Yes	868 (3.9)	315 (2.6)	395 (7.1)	158 (3.5)	
No	21512 (96.1)	11962 (97.4)	5153 (92.9)	4397 (96.5)	
**Heart failure**					<0.001
Yes	663 (3.0)	265 (2.2)	264 (4.8)	134 (2.9)	
No	21717 (97.0)	12012 (97.8)	5284 (95.2)	4421 (97.1)	
**Heart attack**					<0.001
Yes	878 (3.9)	293 (2.4)	375 (6.8)	210 (4.6)	
No	21502 (96.1)	11984 (97.6)	5173 (93.2)	4345 (95.4)	
**Stroke**					<0.001
Yes	769 (3.4)	289 (2.4)	298 (5.4)	182 (4.0)	
No	21611 (96.6)	11988 (97.6)	5250 (94.6)	4373 (96.0)	
**Asthma**					<0.001
Yes	19135 (85.5)	10637 (86.6)	4748 (85.6)	3750 (82.3)	
No	3245 (14.5)	1640 (13.4)	800 (14.4)	805 (17.7)	
**Diabetes**					<0.001
Yes	2730 (12.2)	1362 (11.1)	942 (17.0)	426 (9.4)	
No	19160 (85.6)	10691 (87.1)	4426 (79.8)	4043 (88.8)	
Borderline	490 (2.2)	224 (1.8)	180 (3.2)	86 (1.9)	
**Drinking status**					<0.001
Never drinks	2417 (10.8)	2124 (17.3)	161 (2.9)	132 (2.9)	
Previous drinker	3312 (14.8)	1473 (12.0)	1154 (20.8)	688 (15.1)	
Light and moderate drinker	8146 (36.4)	4874 (39.7)	2208 (39.8)	1043 (22.9)	
Heavy drinker	8505 (38.1)	3806 (31.0)	2025 (36.5)	2692(59.0)	
**BMI** (kg/m^2^), mean ± SD	29.20 ± 6.88	29.29 ± 7.00	29.80 ± 6.63	28.22 ± 6.76	<0.001
**PA**					<0.001
<600	14997 (67.0)	8568 (69.8)	3740 (67.4)	2689 (59.0)	
>1200	6079 (27.2)	2991 (24.4)	1486 (26.8)	1602 (35.2)	
600–1200	1304 (5.8)	718 (5.8)	322 (5.8)	264 (5.8)	
Daily calorie intake, mean ± SD	2120.80 ± 991.18	2055.03 ± 929.56	2117.49 ± 937.08	2302.09 ± 1176.63	<0.001
**Sleep disorders**					<0.001
Yes	5716 (25.5)	2642 (21.5)	1642 (29.6)	1432 (31.4)	
No	16664 (74.5)	9635 (78.5)	3906 (70.4)	3123 (68.6)	
**Sleep duration** (hours)	7.05 ± 2.73	7.08 ± 2.18	7.10 ± 2.56	6.92 ± 3.98	<0.001
**Sleep duration group**					<0.001
Sleep-deprived	8023 (35.8)	4164 (33.9)	1866 (33.6)	1993 (43.8)	
Non-sleep-deprived	14357 (64.2)	8113 (66.1)	3682 (66.4)	2562 (56.2)	

Stratified by smoking status: never smokers (<100 cigarettes lifetime) former smokers (≥100 cigarettes not currently smoking) current smokers (≥100 cigarettes currently smoking). All estimates weighted to account for NHANES survey design. Continuous variables: p-values from weighted linear regression. Categorical variables: p-values from weighted chi-squared tests. PIR poverty income ratio. BMI: body mass index. PA: Physical Activity Index.

the other two groups, which may reflect a tendency for long-term smokers to quit with advancing age. The majority of participants in all groups were non-Hispanic White (non-smoker, 4998; 40.7%; former smoker, 3110; 56.1%; current smoker, 2309; 50.7%). Regarding education level, a higher level of education was significantly more common among non-smokers (some college or higher:7326, 59.7%) and former smokers (some college or higher :3018; 54.4%), suggesting that education level may positively influence smoking cessation or health-related behaviors. Analysis of marital status showed a lower proportion of current smokers among those with a partner. While most health indicators exhibited statistically significant differences across groups, further analysis indicated no significant differences in the prevalence of specific chronic conditions – such as heart disease, asthma, and diabetes – among the three groups. Notably, both former (1642; 29.6%) and current smokers (1432; 31.4%) reported significantly higher rates of sleep disorders compared to never smokers (2642; 21.5%). Additionally, current smokers had a shorter average sleep duration (6.92 ± 3.98 hours) and a significantly higher proportion of individuals with deprived sleep (1993; 43.8%) compared to the other groups.

Table 2 presents the characteristics of participants from NHANES 2005–2020 by tobacco exposure status. Based on tobacco exposure levels, participants were categorized into three groups: No tobacco exposure (n=11732; 52.4%), low tobacco exposure (n=4763; 21.3%), and high tobacco exposure (n=5908; 26.3%). Statistical analysis revealed significant differences (p<0.05) among the groups in terms of age, race, education level, marital status, PRI, and BMI, as detailed in Table 1. No significant difference was observed in gender distribution across the groups. Regarding health status, tobacco exposure level was significantly associated with the prevalence of asthma, cardiac arrest, heart failure, and diabetes (p<0.05). As tobacco exposure increased, the risk of these conditions showed a gradual yet modest rise. In terms of sleep health, the confirmed tobacco exposure group had a significantly higher prevalence of sleep disorders compared to the suspected and no-exposure groups. Similarly, the proportion of individuals with insufficient sleep was also significantly higher in this group.

**Table 2:** Baseline characteristics of participants, by tobacco exposure status, NHANES 2005–2020 (N=22380)

*Characteristics*	*Total* *(N=22380)* *n (%)*	*Serum cotinine level (ng/mL)*			*p*
		*No tobacco* *exposure* *(<0.05)* *(N=11725)* *n (%)*	*Suspected tobacco* *exposure* *(0.05–3)* *(N=4759)* *n (%)*	*Definite tobacco* *exposure* *(>3)* *(N=5896)* *n (%)*	
**Age** (years), mean ± SD	47.28 ± 16.74	50.24 ± 16.75	44.98 ± 17.38	42.79 ± 14.90	<0.001
**Gender**					0.886
Male	12488 (55.8)	6543 (55.8)	2675 (56.2)	3270 (55.5)	
Female	9892 (44.2)	5182 (44.2)	2084 (43.8)	2626 (45.5)	
**Race**					<0.001
Mexican American	1746 (7.8)	1032 (8.8)	381 (8.0)	333 (5.6)	
Non-Hispanic Black	2283 (10.2)	762 (6.5)	719 (15.1)	802 (14.4)	
Non-Hispanic White	15778 (70.5)	8512 (72.6)	3093 (65.0)	4173 (70.2)	
Other Hispanic	1074 (4.8)	610 (5.2)	238 (5.0)	226 (4.0)	
Other	1499 (6.6)	809 (6.9)	328 (6.9)	362 (5.8)	
**Education level**					<0.001
High school	4767 (21.3)	1935 (16.5)	1195 (25.1)	1637 (28.6)	
Lower than high school	3424 (15.3)	1290 (11.0)	871 (18.3)	1263 (22.0)	
Some college or higher	14189 (63.4)	8500 (72.6)	2693 (56.6)	2996 (49.4)	
**Marital status**					<0.001
Married/living with partner	15039 (67.2)	8583 (73.2)	2922 (61.4)	3534 (59.0)	
Never married	2954 (13.2)	1173 (10.0)	833 (17.5)	948 (16.7)	
Widowed/divorced/separated	4387 (19.6)	1969 (16.8)	1004 (21.1)	1414 (24.3)	
**PIR**					<0.001
<2	7564 (33.8)	2920 (24.9)	1946 (40.9)	2698 (47.1)	
≥2	14816 (66.2)	8805 (75.1)	2813 (59.1)	3198 (52.9)	
**Angina pectoris**					0.201
Yes	470 (2.1)	235 (2.0)	90 (1.9)	145 (2.4)	
No	21910 (97.9)	11490 (98.0)	4669 (98.1)	5751 (97.6)	
**Coronary artery disease**					0.549
Yes	716 (3.2)	375 (3.2)	143 (3.0)	206 (3.5)	
No	21664 (96.8)	11350 (96.8)	4616 (97.0)	5690 (96.5)	
**Heart failure**					0.011
Yes	492 (2.2)	223 (1.9)	128 (2.7)	141 (2.4)	
No	21888 (97.8)	11502 (98.1)	4631 (97.3)	5755 (97.6)	
**Heart attack**					<0.001
Yes	694 (3.1)	305 (2.6)	152 (3.2)	237 (3.9)	
No	21686 (96.9)	11420 (97.4)	4607 (96.8)	5659 (96.1)	
**Stroke**					0.266
Yes	582 (2.6)	281 (2.4)	124 (2.6)	177 (2.9)	
No	21798 (97.4)	11444 (97.6)	4635 (97.4)	5719 (97.1)	
**Asthma**					<0.001
Yes	3335 (14.9)	1595 (13.6)	728 (15.3)	1012 (17.5)	
No	19045 (85.1)	10130 (86.4)	4031 (84.7)	4884 (82.5)	
**Diabetes**					0.002
Yes	2036 (9.0)	1090 (9.3)	585 (10.4)	361 (7.5)	
No	20344 (89.0)	10635 (88.6)	4174 (87.7)	5535 (90.7)	
**Drinking status**					<0.001
Never drinks	2417 (10.8)	1607 (13.7)	595 (12.5)	215 (3.3)	
Previous drinker	3312 (14.8)	1770 (15.1)	662 (13.9)	880 (15.0)	
Light and moderate drinker	8146 (36.4)	5065 (43.2)	1542 (32.4)	1539 (25.0)	
Heavy drinker	8505 (38.1)	3283 (28.0)	1960 (41.2)	3262 (56.7)	
**BMI** (kg/m^2^), mean ± SD	29.20 ±6.88	29.11 ±6.57	30.36±7.52	28.44±6.81	<0.001
**PA**					<0.001
<600	14457 (64.6)	8079 (68.9)	2993 (62.9)	3385 (57.2)	
>1200	6401 (28.6)	2861 (24.4)	1399 (29.4)	2141 (36.5)	
600–1200	1522 (6.8)	785 (6.7)	367 (7.7)	370 (6.4)	
**Daily calorie intake**, mean ± SD	2161.03 ± 967.82	2074.78 ± 860.81	2193.41 ± 983.61	2316.69 ± 1131.70	<0.001
**Sleep disorders**					<0.001
Yes	6177 (27.6)	3060 (26.1)	1185 (24.9)	1932 (32.5)	
No	16203 (72.4)	8665 (73.9)	3574 (75.1)	3964 (67.5)	
**Sleep duration** (hours)	7.12 ± 2.47	7.19 ± 1.81	7.11 ± 2.79	6.98 ± 3.27	<0.001
**Sleep duration group**					
Sleep-deprived	7229 (32.3)	3306 (28.2)	1566 (32.9)	2357 (40.5)	<0.001
Non-sleep-deprived	15151 (67.7)	8419 (71.8)	3193 (67.1)	3539 (59.5)	

All estimates weighted to account for NHANES survey design. Continuous variables: p-values from weighted linear regression. Categorical variables: p-values from weighted chi-squared tests. PIR: poverty income ratio. BMI: body mass index. PA: Physical Activity Index.

*The association between smoking status and the risk of sleep disorders/sleep deprivation in NHANES*

As presented in Table 3. In the analysis of sleep disorder risk, both former and current smokers showed significantly elevated risks compared to non-smokers, and these associations remained statistically significant across models with varying degrees of adjustment. After multivariable adjustment (adjusted for age, gender, race, education, marital status, PIR, BMI, heart failure, heart attack, asthma, diabetes, drinking status, daily calorie intake, MET), former smokers had a 35% higher risk of sleep disorders (AOR=1.35; 95% CI: 1.21–1.50, p<0.001), while current smokers had a 68% higher risk (AOR=1.68; 95% CI: 1.49–1.90, p<0.001). Regarding sleep duration, current smokers exhibited a significantly higher risk of deprived sleep (<7 hours per day) compared to the other groups (Table 4). This association remained significant after multivariable adjustment (adjusted for age, gender, race, education level, marital status, pir, BMI, heart failure, heart attack, asthma, diabetes, drinking status, daily calorie intake, MET) (AOR=1.52; 95% CI: 1.36–1.70, p<0.001).

**Table 3:** Association between smoking status/serum cotinine concentration and the risk of sleep disorder/sleep deprivation, NHANES, 2005–2020 (N=22380)

*Exposure*	*Outcome*	*Crude Model* *OR (95% CI) p*	*Model 1* *AOR (95% CI) p*	*Model 2 AOR* *(95% CI) p*
**Smoking status**	**Sleep deprivation**			
Never smoked (ref.)				
Former smoker		0.99 (0.90–1.09) 0.841	1.08 (0.98–1.19) 0.139	1.02 (0.92–1.14) 0.646
Current smoker		1.64 (1.49–1.81) <0.001	1.55 (1.39–1.52) <0.001	1.52 (1.36–1.70) <0.001
p for trend		<0.001	<0.001	<0.001
**Smoking status**	**Sleep disorder**			
Never smoked (ref.)				
Former smoker		1.66 (1.50–1.84) <0.001	1.44 (1.29–1.60) <0.001	1.35 (1.20–1.51) <0.001
Current smoker		1.69 (1.52–1.88) <0.001	1.69 (1.51–1.69) <0.001	1.68 (1.49–1.90) <0.001
p for trend		<0.001	<0.001	<0.001
**Serum cotinine level**	**Sleep deprivation**			
T1 (ref.)				
T2		0.94 (0.84–1.05) <0.001	1.00 (0.89–1.13) <0.001	0.95 (0.84–1.07) <0.001
T3		1.36 (1.23–1.50) <0.001	1.48 (1.33–1.65) <0.001	1.44 (1.29–1.62) <0.001
p for trend		<0.001	<0.001	<0.001
**Serum cotinine level**				
Continuous		1.04 (1.03–1.05) <0.001	1.05 (1.03–1.06) <0.001	1.04 (1.03–1.06) <0.001
p for trend		<0.001	<0.001	<0.001
**Serum cotinine level**	**Sleep disorder**			
T1 (ref.)				
T2		1.24 (1.13–1.38) <0.001	1.11 (1.00–1.23) <0.001	1.07 (0.97–1.19) <0.001
T3		1.73 (1.58–1.90) <0.001	1.55 (1.40–1.71) <0.001	1.52 (1.36–1.69) <0.001
p for trend		<0.001	<0.001	<0.001
**Serum cotinine level**				
Continuous		1.07 (1.05–1.08) <0.001	1.05 (1.04–1.07) <0.001	1.05 (1.04–1.06) <0.001
p for trend		<0.001	<0.001	<0.001

Crude Model: unadjusted. AOR: adjusted odds ratio. Model 1: adjusted for age, gender, race, education level, marital status, and PIR. Model 2: adjusted as for Model 1 plus BMI, heart failure, heart attack, asthma, diabetes, drinking status, daily calorie intake, and MET. Odds ratios (95% CI) for the associations between smoking status/serum cotinine concentration and the risk of sleep disorder/sleep deprivation, estimated using weighted linear regression and weighted logistic regression models. All models accounted for NHANES survey weights. T1: <0.05 ng/mL. T2: 0.05–3 ng/mL. T3: >3 ng/mL (tertiles).

**Table 4:** Threshold effect analysis and piecewise regression analysis of serum cotinine continuous concentration and the risk of sleep deprivation/sleep disorder, NHANES, 2005–2020 (N=22380)

*Exposure*	*Outcome*	*Crude Model* *OR (95% CI) p*	*Model 1* *AOR (95% CI) p*	*Model 2* *AOR (95% CI) p*
**ln (serum cotinine) < -1.198**	**Sleep deprivation**	1.19 (1.13–1.26) <0.001	1.12 (1.07–1.21) <0.001	1.09 (1.03–1.26) 0.002
p for trend		<0.001	<0.001	<0.001
**-1.198 ≤ ln (serum cotinine) <3.995**		1.01 (0.95–1.08) 0.731	1.01 (0.94–1.08) 0.788	1.02 (0.95–1.09) 0.602
p for trend		<0.001	<0.001	<0.001
**ln (serum cotinine) ≥3.995**		1.30 (1.12–1.49) <0.001	1.32 (1.14–1.54) <0.001	1.33 (1.15–1.55) <0.001
p for trend		<0.001	<0.001	<0.001
**ln (serum cotinine) < -0.198**	**Sleep disorder**	1.01 (0.96–1.06) 0.676	1.06 (0.99–1.11) 0.038	1.02 (0.97–1.08) 0.415
p for trend		<0.001	<0.001	<0.001
**ln (serum cotinine) ≥ -0.198**		1.13 (1.08–1.18) <0.001	1.09 (1.04–1.14) <0.001	1.08 (1.02–1.14) 0.008
p for trend		<0.001	<0.001	<0.001

AOR: adjusted odds ratio. Crude Model: unadjusted. Model 1: adjusted for age, gender, race, education level, marital status, and PIR. Model 2: adjusted as for Model 1 plus BMI, heart failure, heart attack, asthma, diabetes, drinking status, daily calorie intake, and MET. Odds ratios (95% CI) for the associations between smoking status/serum cotinine concentration and the risk of sleep disorder/sleep deprivation, estimated using weighted linear regression and weighted logistic regression models. All models accounted for NHANES survey weights. Two critical thresholds were identified for sleep deprivation: ln (serum cotinine) = -1.198 and 3.995. The risk of sleep deprivation increased significantly with higher serum cotinine levels when ln (serum cotinine) >3.995. One critical threshold was identified for sleep disorder: ln (serum cotinine) = -0.198; above this value, the risk of sleep disorder increased more rapidly with rising serum cotinine levels.

*The association between serum cotinine concentration and the risk of sleep disorders/sleep deprivation in NHANES*

As shown in Table 3, in the analysis of sleep disorder risk, a significant association was observed between high tobacco exposure and increased risk of sleep disorders compared to the no-exposure group, and this association remained consistent across models with varying levels of adjustment [Crude Model: OR=1.36; 95% CI: 1.23–1.50, p<0.001; Model 1 (partially adjusted: adjusted for age, gender, race, education level, marital status, PIR): AOR=1.48; 95% CI: 1.33–1.65, p<0.001; Model 2 (fully adjusted: as for Model 1plus adjusted for BMI, heart failure, heart attack, asthma, diabetes, drinking status, daily calorie intake, and PA): AOR=1.44; 95% CI: 1.29–1.62, p<0.001]. In contrast, no statistically significant association was found between suspected tobacco exposure and sleep disorder risk in any of the models [Crude Model: OR=0.94; 95% CI: 0.84–1.05, p=0.242; Model 1: AOR=1.00; 95% CI: 0.89–1.13, p=0.953; Model 2: AOR=0.95; 95% CI: 0.84–1.07, p=0.385].

When serum cotinine concentration was analyzed as a continuous variable (after natural log transformation), a significant positive association was observed with the risk of sleep disorders. Across all models, each unit increase in ln-transformed cotinine concentration was associated with a statistically significant elevation in sleep disorder risk [Crude Model: OR=1.04; 95% CI: 1.03–1.05, p<0.001; Model 1: AOR=1.05, 95% CI: 1.03–1.06, p<0.001; Model 2: AOR=1.04, 95% CI: 1.03–1.06, p<0.001]. Based on the estimates from Model 2, a 2.83 ng/mL increase in serum cotinine concentration – equivalent to a one-unit change on the ln scale – corresponded to an approximately 4% increase in the risk of sleep disorders.

A similar trend (Table 3) was observed in the analysis of deprived sleep risk. High exposure was significantly associated with an increased risk of deprived sleep [Crude Model: OR=1.73; 95% CI: 1.58–1.90, p<0.001; Model 1: AOR=1.55; 95% CI: 1.40–1.71, p<0.001; Model 2: AOR=1.52; 95% CI: 1.36–1.69, p<0.001]. In contrast, the low exposure group did not exhibit a consistently significant association [Crude Model: OR=1.24; 95% CI: 1.13–1.38, p<0.001; Model 1: AOR=1.11; 95% CI: 0.97–1.19, p=0.06; Model 2: AOR=1.07; 95% CI: 0.97–1.19, p=0.183]. When analyzed as a continuous variable, each unit increase in ln-transformed cotinine concentration was also significantly associated with higher risk of deprived sleep across all models [Crude Model: OR=1.07; 95% CI: 1.05–1.08, p<0.001; Model 1: AOR=1.05; 95% CI: 1.04–1.07, p<0.001; Model 2: AOR=1.05; 95% CI: 1.04–1.06, p<0.001].

*Threshold effect and restricted cubic spline analyses of the association between serum cotinine and sleep disorder/sleep deprivation risk*

**Figure 1:**
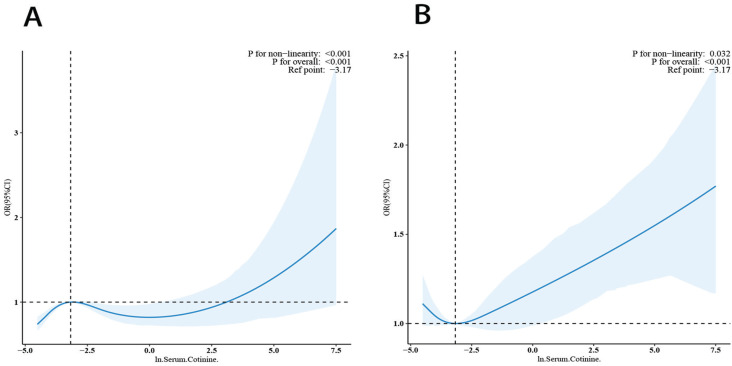
Association between serum cotinine concentration and risk of sleep deprivation (A), and risk of sleep disorder (B). Odds ratio (95% CI) shows a U-shaped relationship between risk and log-transformed serum cotinine (ng/mL), obtained using restricted cubic spline regression adjusted for covariates (p for nonlinearity <0.001)

We employed restricted cubic spline (RCS) analysis to examine the nonlinear relationship between natural log-transformed serum cotinine levels and the risks of sleep disorder and sleep deprivation. As shown in Figure 1, significant nonlinear associations were observed for both outcomes (sleep disorder: p for nonlinearity =0.032; sleep deprivation: p for nonlinearity <0.001).

Based on these findings, we further conducted threshold effect analysis and established piecewise regression models. The results (Table 4) indicated that for sleep deprivation risk, when log-transformed serum cotinine was < -1.196 (serum cotinine level was 0.302 ng/mL), each unit increase was associated with a significant 9% rise in risk (AOR=1.09; 95% CI: 1.03–1.26, p<0.001). No significant association was observed between -1.196 and 3.995 (p>0.05), whereas beyond 3.995 (serum cotinine level was 54.33 ng/mL), each unit increase led to a significant 33% increase in risk (AOR=1.33; 95% CI: 1.15–1.55, p<0.001).

A similar pattern was observed for sleep disorder risk (Table 4): no significant association was found when log-transformed serum cotinine was < -0.198 (serum cotinine level was 0.820 ng/mL), but beyond this threshold, the risk increased significantly (AOR=1.08; 95% CI: 1.02–1.14, p<0.001).

*Mendelian randomization analysis results Smoking status and sleep disorders/sleep deprivation*

After a rigorous selection process, genetic instruments associated with smoking status were obtained from the EBI database, including 84 SNPs for never smoking, 40 for former smoking, and 27 for current smoking. All SNPs exhibited F-statistics greater than 10, effectively mitigating weak instrument bias. Following the removal of palindromic structures, 78 SNPs for never smoking, 38 for former smoking, and 27 for current smoking were retained for subsequent analyses (Supplementary file Tables 1–3).

**Figure 2:**
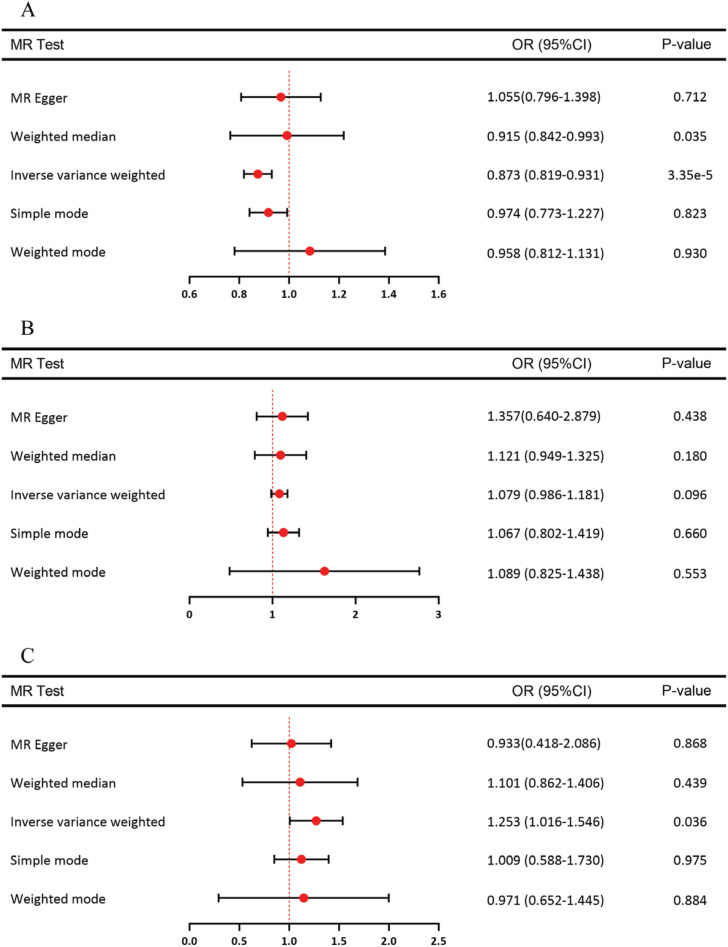
Mendelian randomization analyses using GWAS summary data of the association between smoking status and risk of sleep deprivation: A) non-smoker, B) former smoker, and C) current smoker

MR analysis results (Figures 2A–2C) indicated that the IVW method suggested a significant inverse relationship between genetic predisposition to never smoking and the risk of deprived sleep (OR=0.870; 95% CI: 0.819–0.931, p=3.30×10^-5^). A positive yet statistically non-significant association was observed between former smoking and deprived sleep (OR=1.079; 95% CI: 0.986–1.181, p=0.096). In contrast, a significant positive relationship was identified between current smoking and the risk of deprived sleep (OR=1.253; 95% CI: 1.015–1.548, p=0.036).

**Figure 3:**
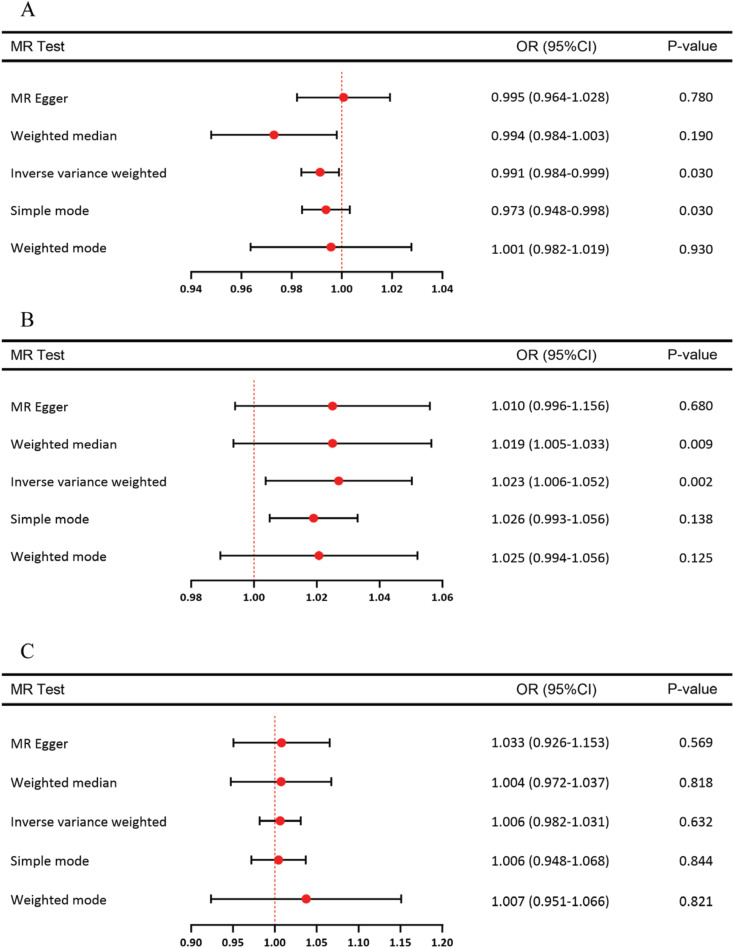
Mendelian randomization analyses using GWAS summary data of the association between smoking status and risk of sleep disorder: A) non-smoker, B) former smoker, and C) current smoker

In the analysis with sleep disorders as the outcome, the same selection strategy was applied, incorporating 78 SNPs for never smoking, 38 for former smoking, and 27 for current smoking (Supplementary file Tables 4–6). Mendelian randomization analysis results (Figures 3A–3C) indicated that IVW analysis indicated a significant inverse association between genetic predisposition to never smoking and the risk of sleep disorders (OR=0.992; 95% CI: 0.985–0.999, p=0.027). Former smoking showed a significant positive association with sleep disorder risk (OR=1.015; 95% CI: 1.005–1.026, p=0.003). In contrast, no significant relationship was observed between current smoking and sleep disorders (OR=1.004; 95% CI: 0.985–1.023, p=0.701).

No significant bias was detected in the tests for heterogeneity or horizontal pleiotropy (Supplementary file Table 9). To further validate the robustness of the results, we conducted supplementary analyses including funnel plots, leave-one-out sensitivity analysis, forest plots, and scatter plots (Supplementary file Figures 3–8). All auxiliary analyses consistently supported the main conclusions, indicating that the findings of this study are highly reliable.

*Serum cotinine and sleep disorders/sleep deprivation*

After a rigorous selection process, we identified 11 eligible SNPs from the EBI database as instrumental variables. All SNPs exhibited F-statistics greater than 10 (Supplementary file Tables 7 and 8), effectively minimizing potential bias from weak instruments.

**Figure 4:**
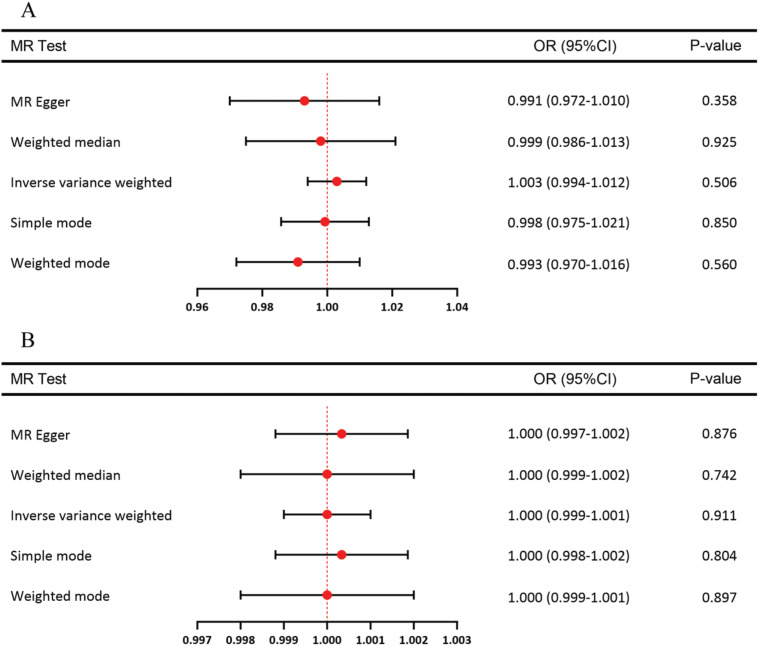
Mendelian randomization analysis using GWAS summary data of the association between serum cotinine concentration and risk of sleep deprivation (A), and risk of sleep disorder (B)

MR analysis results (Figure 4A) indicated that the IVW method did not reveal a significant relationship between serum cotinine levels and the risk of deprived sleep (OR=1.000; 95% CI: 0.999–1.001, p=0.911). Similarly, no significant association (Figure 4B) was observed between serum cotinine and the risk of sleep disorders (OR=1.003; 95% CI: 0.994–1.012, p=0.506).

Further tests for heterogeneity and horizontal pleiotropy indicated no significant bias in the results (Supplementary file Table 9). To validate the robustness of the MR findings, we generated funnel plots, leave-one-out sensitivity analysis plots, forest plots, and scatter plots (Supplementary file Figures 9 and 10). All sensitivity analyses consistently supported the null findings, indicating high reliability of the conclusions in this study.

## DISCUSSION

The relationship between smoking and sleep disorders remains a subject of ongoing academic debate; however, numerous studies across diverse populations and types of exposure have demonstrated a complex association between tobacco use and sleep disturbances^20,21^. For example, a large cross-sectional study among young women in Japan revealed that long-term exposure to secondhand smoke was significantly associated not only with poorer subjective sleep quality but also with a potential increase in the risk of nocturnal bruxism^22^. These findings imply that even non-active smokers may experience disruptions in sleep architecture – potentially mediated by neurostimulatory or anxiety-related pathways – leading to impaired sleep continuity and reduced restorative function. Moreover, research in pediatric populations further supports the broad significance of this association^23^. A nationally representative survey in the United States demonstrated that tobacco exposure, whether active or passive, was significantly correlated with shorter sleep duration, difficulties initiating sleep, and more frequent nighttime awakenings among school-aged children. These effects may be partly explained by nicotine’s action as a central nervous system stimulant, which can dysregulate sleep–wake cycles^24^. Particularly during critical neurodevelopmental stages, toxic components in tobacco may disrupt melatonin secretion and destabilize circadian rhythmicity^25^.

Although a strict dose-response relationship between smoking intensity and sleep disturbances has not been fully elucidated, accumulating evidence strongly indicates that tobacco exposure – whether active or passive – is a significant risk factor for impaired sleep quality^6^. Nicotine, the primary psychoactive component in tobacco, acts as a central nervous system stimulant^26,27^. It binds to and activates nicotinic acetylcholine receptors (nAChRs), triggering the release of neurotransmitters such as dopamine, norepinephrine, serotonin, and glutamate. While these neurochemical alterations may transiently enhance alertness and cognitive performance, they also disrupt both sleep initiation and maintenance. Elevated norepinephrine levels, in particular, can induce a sympathomimetic ‘fight-or-flight’ state that counteracts the parasympathetic activity necessary for restorative sleep^28-30^. Substantial evidence indicates that nicotine exposure adversely alters sleep architecture^31,32^, leading to increased sleep fragmentation, prolonged light sleep (N1 stage), and reductions in both slow-wave sleep (N3) and rapid eye movement (REM) sleep. REM suppression is especially prominent, likely resulting from nicotine’s modulation of cholinergic pathways. A community-based cohort study of over 1000 adults further demonstrated that smokers had more than twice the risk of difficulties falling and staying asleep compared to non-smokers^33^. Polysomnographic (PSG) studies corroborate these findings, showing that nicotine administration prolongs sleep onset latency, reduces REM duration, and increases alpha EEG intrusion during sleep^34^ – a pattern indicative of light, non-restorative sleep. In summary, nicotine disrupts sleep physiology through multi-receptor and multi-transmitter mechanisms, predominantly impairing sleep continuity and deep sleep stages. These pathways provide a neurobiological basis for the association between tobacco exposure and sleep disorders.

Based on data from the NHANES (2005–2020), this study initially used smoking status as the exposure factor and also incorporated serum cotinine as an objective biomarker to systematically evaluate their associations with deprived sleep and sleep disorders. In contrast to previous studies relying on self-reported smoking behavior, this study innovatively combined large-scale secondary dataset analysis with MR methods, effectively reducing subjective reporting bias through the use of an objective biomarker. Secondary dataset analysis showed that, after adjusting for multiple confounding factors, current smoking status was significantly positively associated with the risks of both insufficient sleep and sleep disorders. Analyses treating serum cotinine both as a continuous and a categorical variable consistently indicated a significant positive correlation with the risk of sleep disorders, and this association was particularly pronounced in the high tobacco exposure group. Dose-response analysis further suggested a clear threshold effect of serum cotinine on sleep health, with sleep risks increasing significantly beyond specific concentration levels. Subgroup analyses revealed that the association between smoking behavior and sleep outcomes was not significant in underweight individuals, whereas the association between serum cotinine and sleep disorders was statistically significant only in the obese population. This suggests that obesity may play an effect-modifying role in the relationship between tobacco exposure and sleep, providing new insights into the heterogeneity of tobacco exposure effects across different populations.

In the MR analysis, no significant genetically predicted relationship was observed between current smoking or serum cotinine and either insufficient sleep or sleep disorders. The instrumental variables showed no notable heterogeneity or horizontal pleiotropy, supporting the validity of the results. The discrepancy between the observational and MR findings may be attributed to several factors. First, although multiple covariates were adjusted for in the observational analyses, residual confounding – such as socioeconomic status, psychological stress, or environmental factors – may still have influenced the results. Second, the GWAS data on serum cotinine used in the MR analysis were primarily derived from general populations. The limited number of instrumental variables (11 SNPs) and their relatively low average F-statistics may have constrained statistical power to detect true causal effects, particularly in highly exposed subgroups^35^. Furthermore, neither the sleep disorder nor the cotinine GWAS data were stratified by key modifiers such as obesity, which was suggested in the observational part of this study as a potential effect modifier. Finally, the observational data came from the U.S. NHANES population, while the GWAS data were largely based on European cohorts. Differences in genetic background, smoking behaviors, and sociocultural factors between these populations may also contribute to the inconsistent results.

### Limitations

This study has several limitations. The NHANES analysis employed a cross-sectional design, limiting causal inference. Sleep outcomes were self-reported, which may introduce recall bias. Residual confounding cannot be ruled out despite covariate adjustment. Additionally, population differences between NHANES and the MR datasets may contribute to heterogeneity. These limitations should be considered when interpreting the findings.

## CONCLUSIONS

This study confirmed significant associations between smoking status, serum cotinine levels, and the odds of insufficient sleep and sleep disorders, with a dose-response relationship observed. The effects became significantly stronger when serum cotinine levels reached specific thresholds. However, MR analyses only supported the following: never smokers showed a negative association with insufficient sleep and sleep disorders, former smoking was positively associated with insufficient sleep, and current smoking was positively associated with sleep disorders. No relationship was found between serum cotinine and either insufficient sleep or sleep disorders. Future research should include larger scale genetic studies involving highly exposed populations and diverse ethnic backgrounds, as well as promote stratified MR analyses focusing on high-risk subgroups such as individuals with obesity, to further explore potential mechanisms underlying the association between tobacco exposure and sleep health.

## Supplementary Material



## Data Availability

The data supporting this research are available from the authors on reasonable request.
