# Supplementary material for: Tobacco smoke exposure and sleep problems: A secondary dataset analysis of NHANES and Mendelian randomization analyses
Source: Tob Induc Dis. 2026 Aug 1;24:10.18332/tid/220984. doi: 10.18332/tid/220984 (PMC13450132; doi:10.18332/tid/220984)

Tobacco Induced Diseases

Supplementary file

© 2026 Zhu X. and Jiang C.

DOI:
10.18332/tid/220984

The content has been provided by the author(s) and has not been reviewed, verified, or endorsed by European Publishing. It may not have undergone peer review. The views, opinions, and recommendations expressed are solely those of the author(s) and do not necessarily reflect the position of European Publishing. European Publishing accepts no responsibility or liability for any consequences arising from the use of, or reliance on, this content.

Supplementary Figure1: Participant selection criteria and sample sizes from the NHANES 2005–2020 cycles (final N = 22,380) :Data were derived from seven NHANES cycles (2005–2020). Inclusion criteria: complete data on smoking status, serum cotinine, sleep disorder, sleep duration, and covariates. Exclusion criteria: missing smoking status/cotinine; incomplete sleep data; missing covariates; cancer diagnosis; or current pregnancy

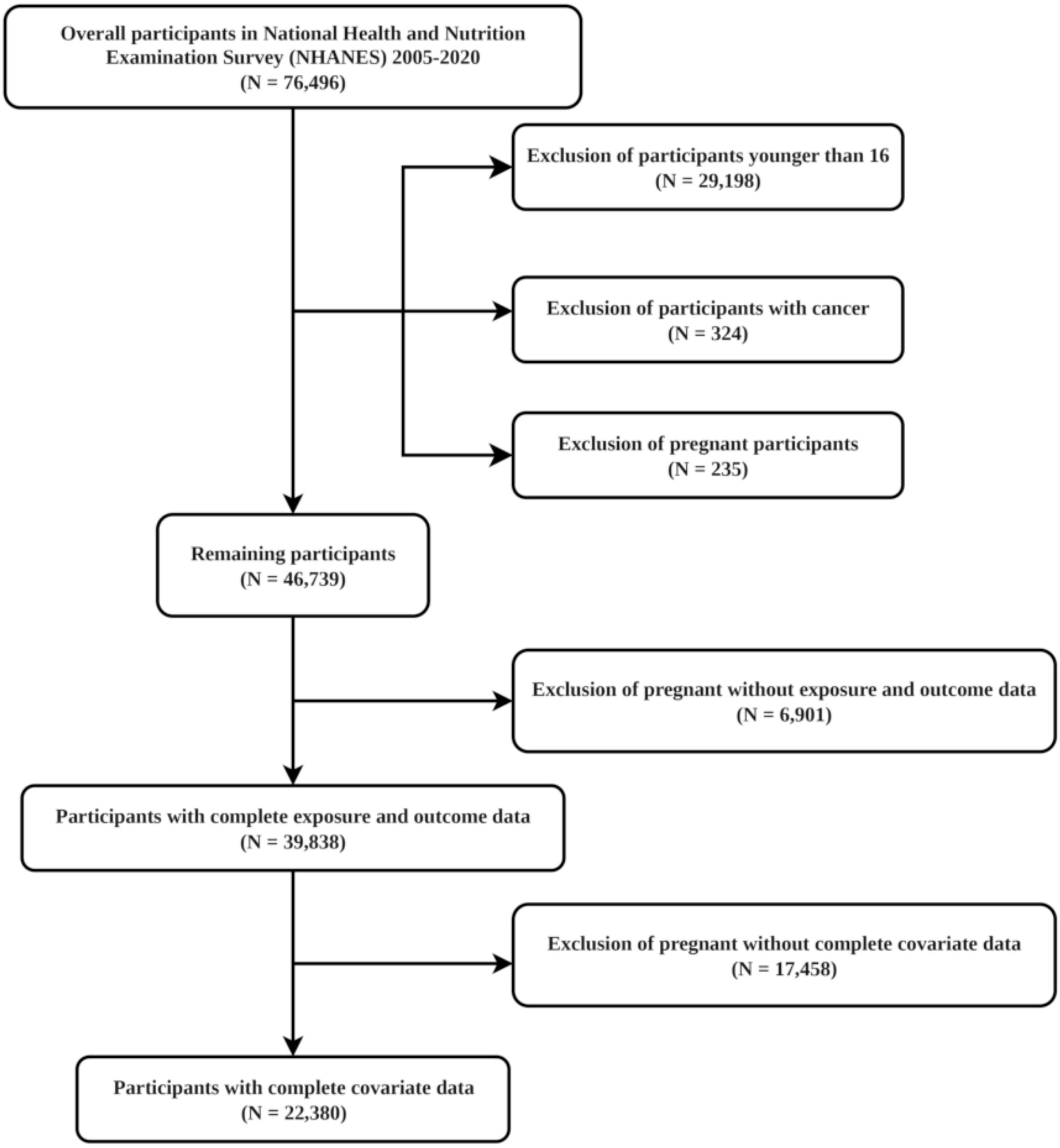

Supplementary Figure2: The overall workflow of the MR analysis:The three core assumptions of Mendelian randomization: (1) relevance—genetic variants are strongly associated with smoking status and serum cotinine; (2) independence—instruments are not associated with confounders; and (3) exclusion restriction—instruments affect sleep outcomes only through smoking status and serum cotinine. These assumptions are essential for valid causal inference in MR analysis.

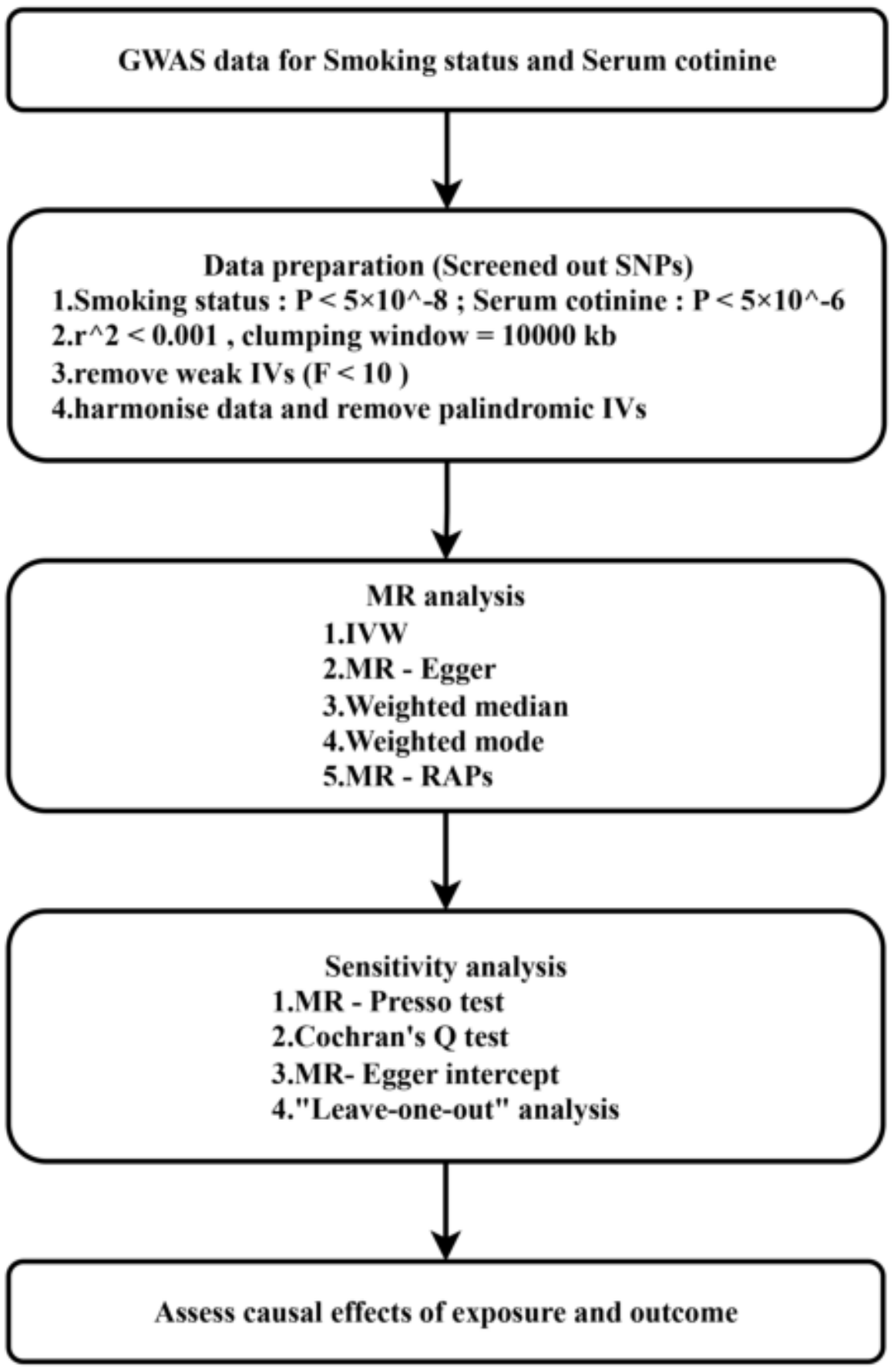

Supplementary Figure3: Supplementary Figure 3: funnel plots, leave-one-out sensitivity analysis, forest plots, and scatter plots(MR analysis of Non smoker and Undersleepers)

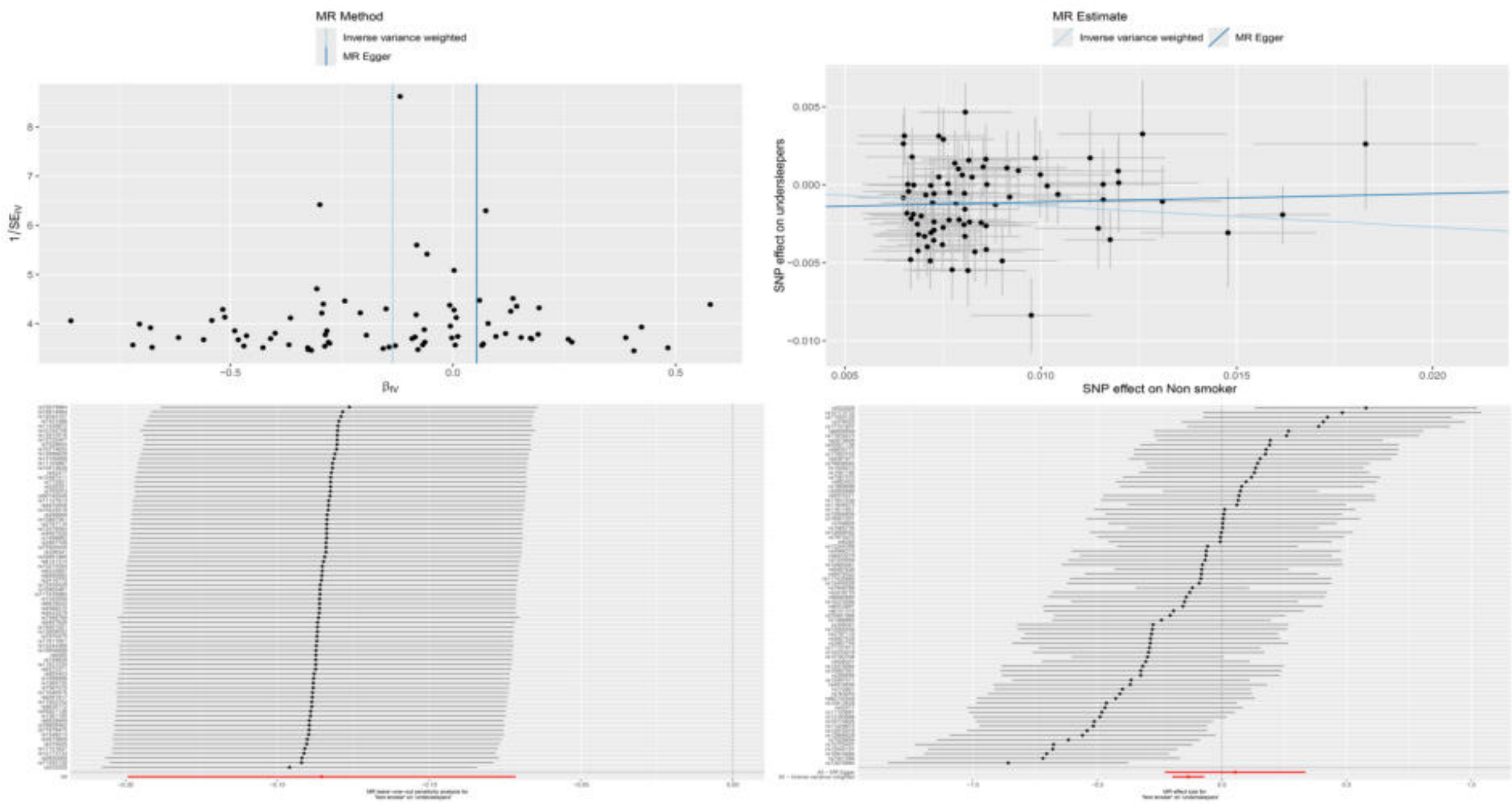

Supplementary Figure4: Supplementary Figure 4: funnel plots, leave-one-out sensitivity analysis, forest plots, and scatter plots(MR analysis of Former smoker and Undersleepers)

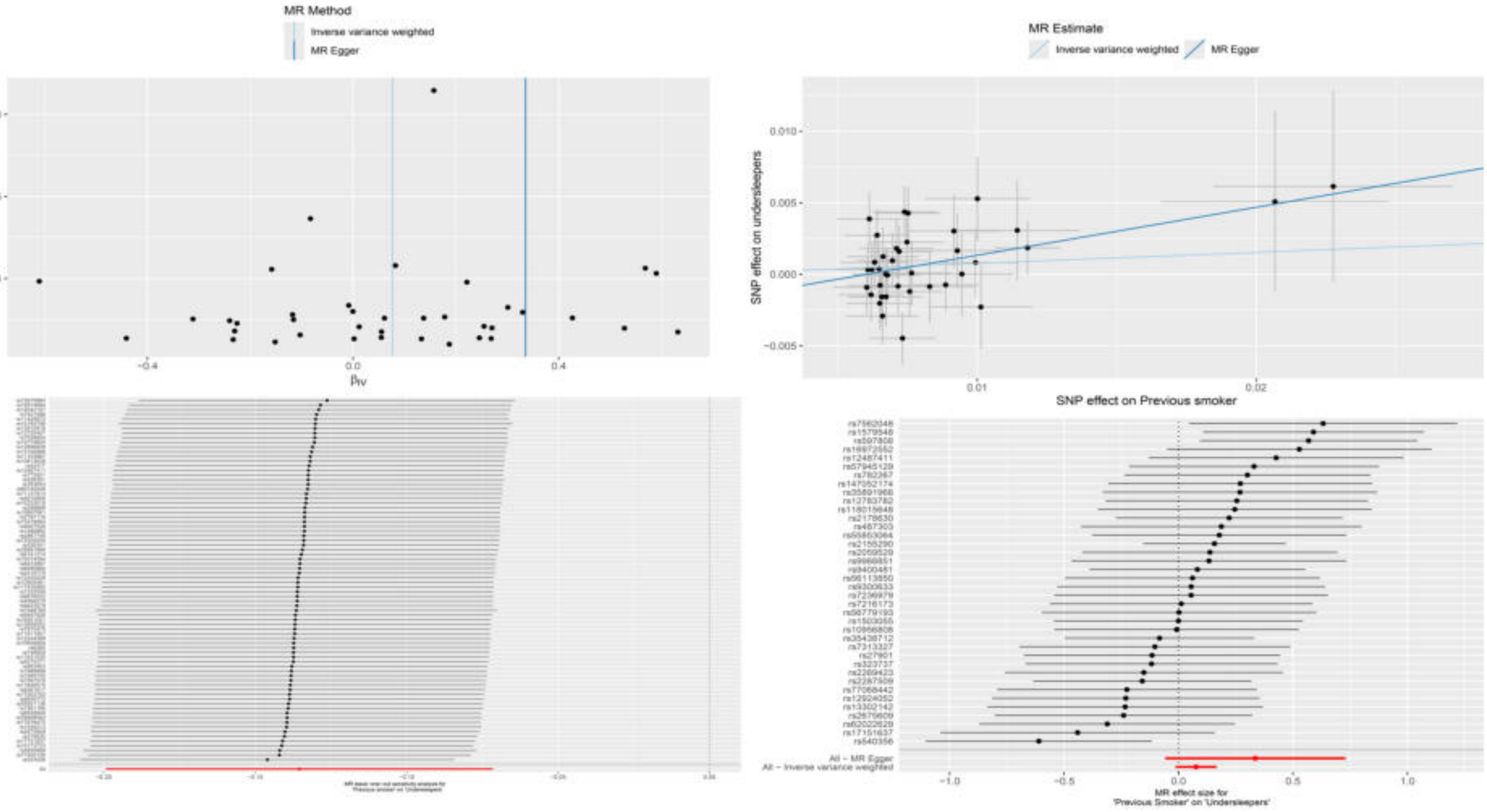

Supplementary Figure5: Supplementary Figure 5: funnel plots, leave-one-out sensitivity analysis, forest plots, and scatter plots(MR analysis of Current smoker and Undersleepers)

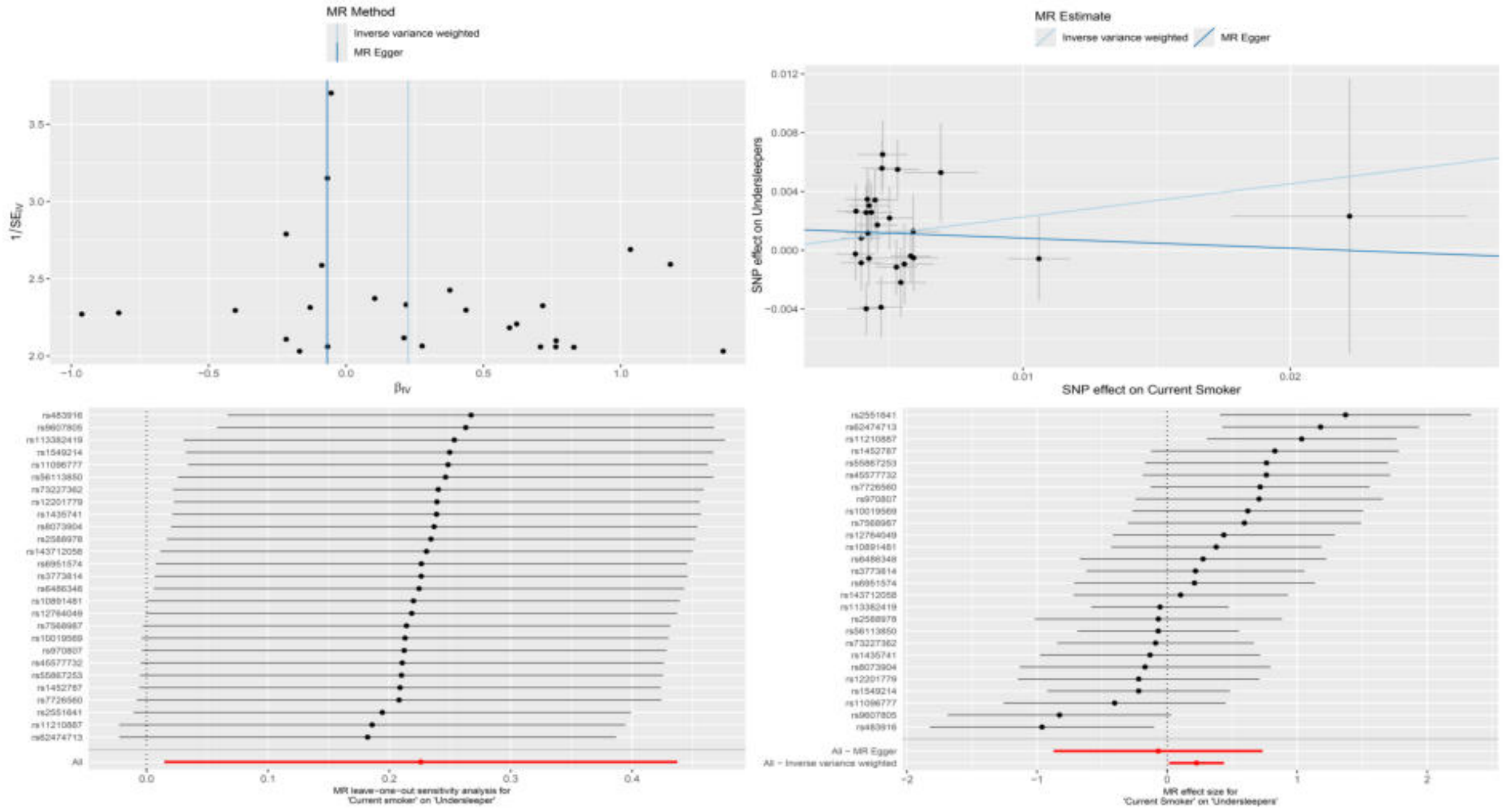

Supplementary Figure6: Supplementary Figure 6: funnel plots, leave-one-out sensitivity analysis, forest plots, and scatter plots(MR analysis of Non smoker and Sleep disorder)

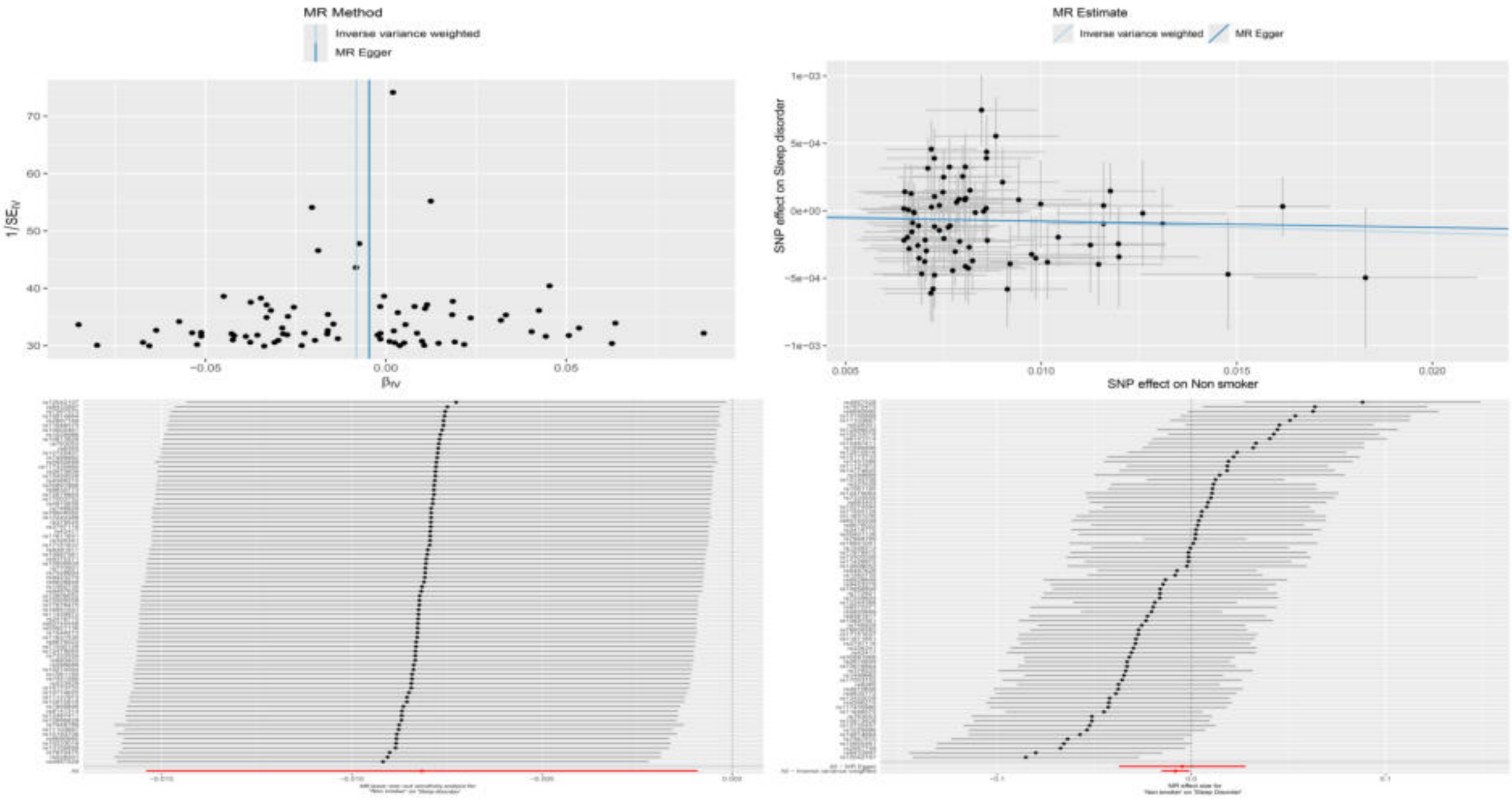

Supplementary Figure7: Supplementary Figure 7: funnel plots, leave-one-out sensitivity analysis, forest plots, and scatter plots(MR analysis of Former smoker and Sleep disorder)

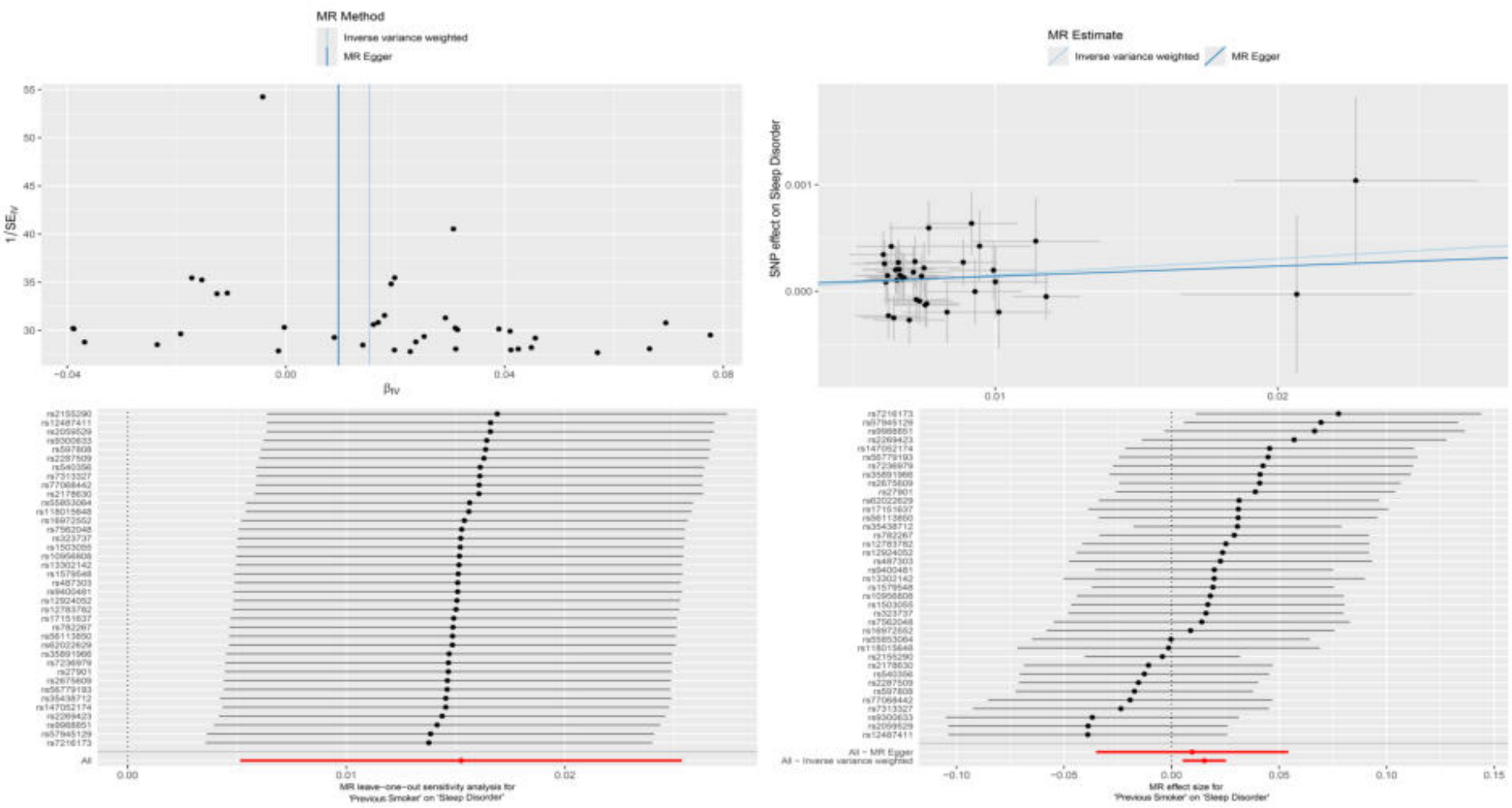

Supplementary Figure8: Supplementary Figure 8: funnel plots, leave-one-out sensitivity analysis, forest plots, and scatter plots(MR analysis of Current smoker and Sleep disorder)

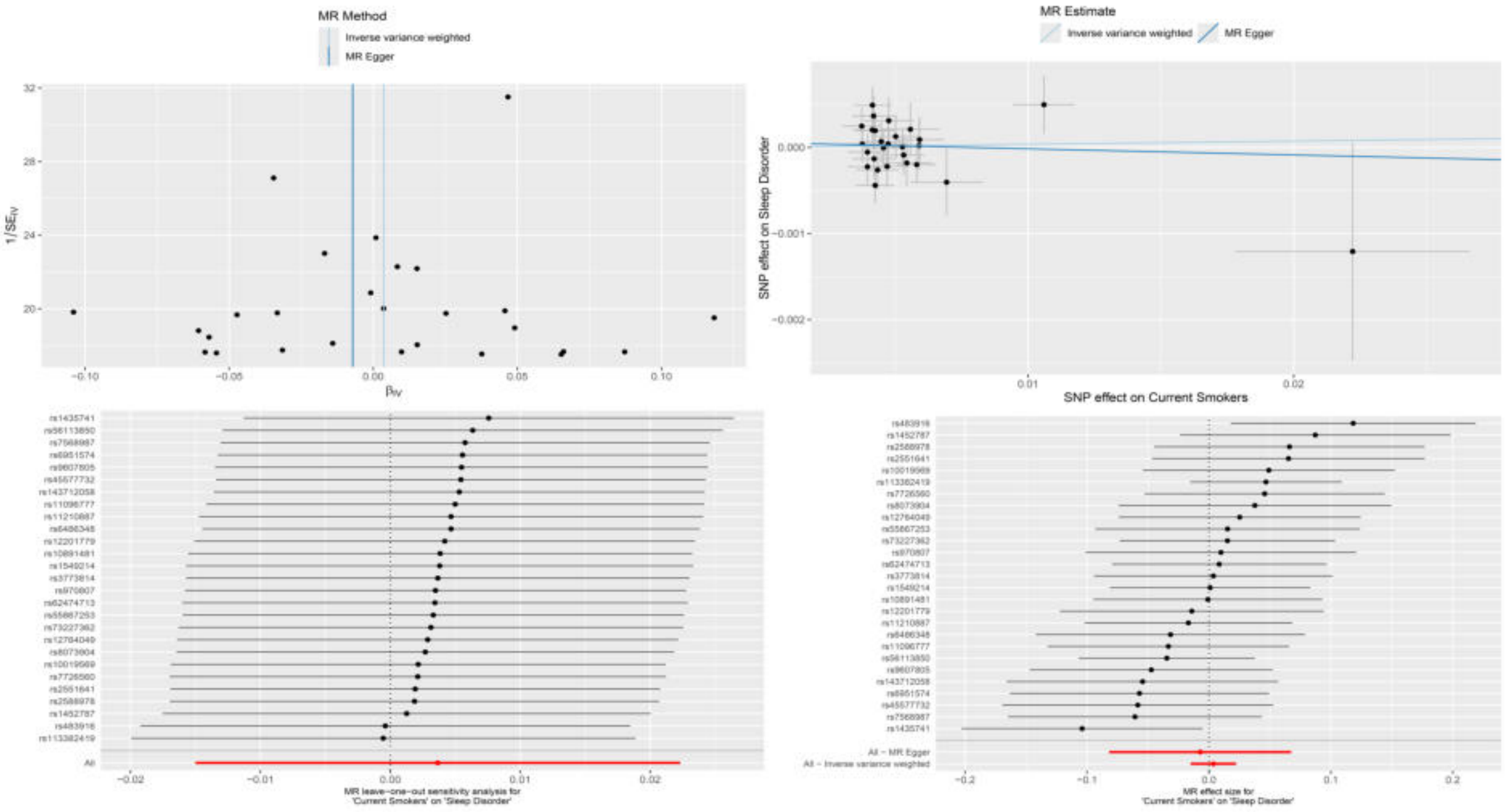

Supplementary Figure9: Supplementary Figure 9: funnel plots, leave-one-out sensitivity analysis, forest plots, and scatter plots(MR analysis of Serum Cotinine and Undersleepers)

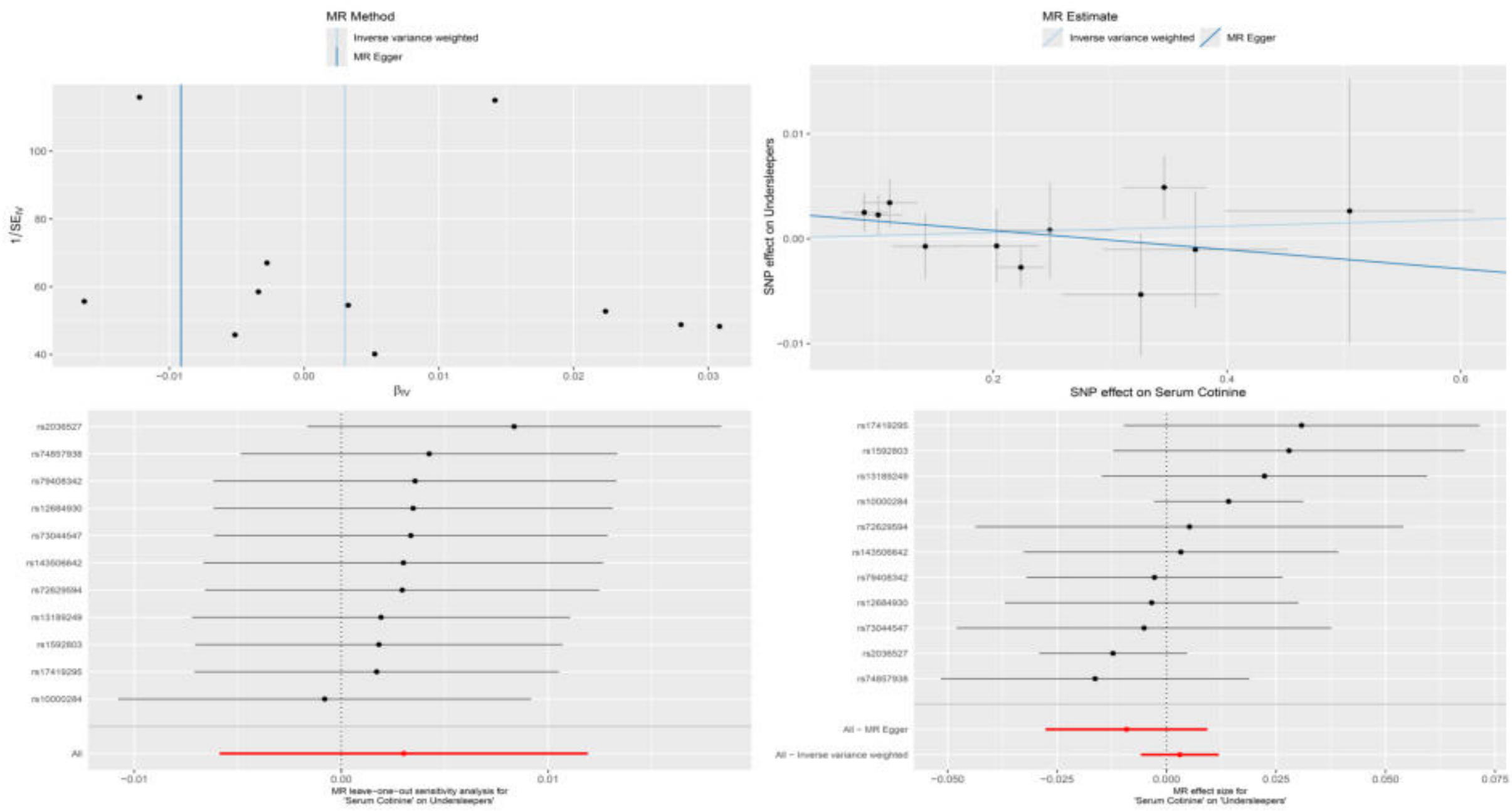

Supplementary Figure10: Supplementary Figure 10: funnel plots, leave-one-out sensitivity analysis, forest plots, and scatter plots(MR analysis of Serum Cotinine and Sleep disorder)

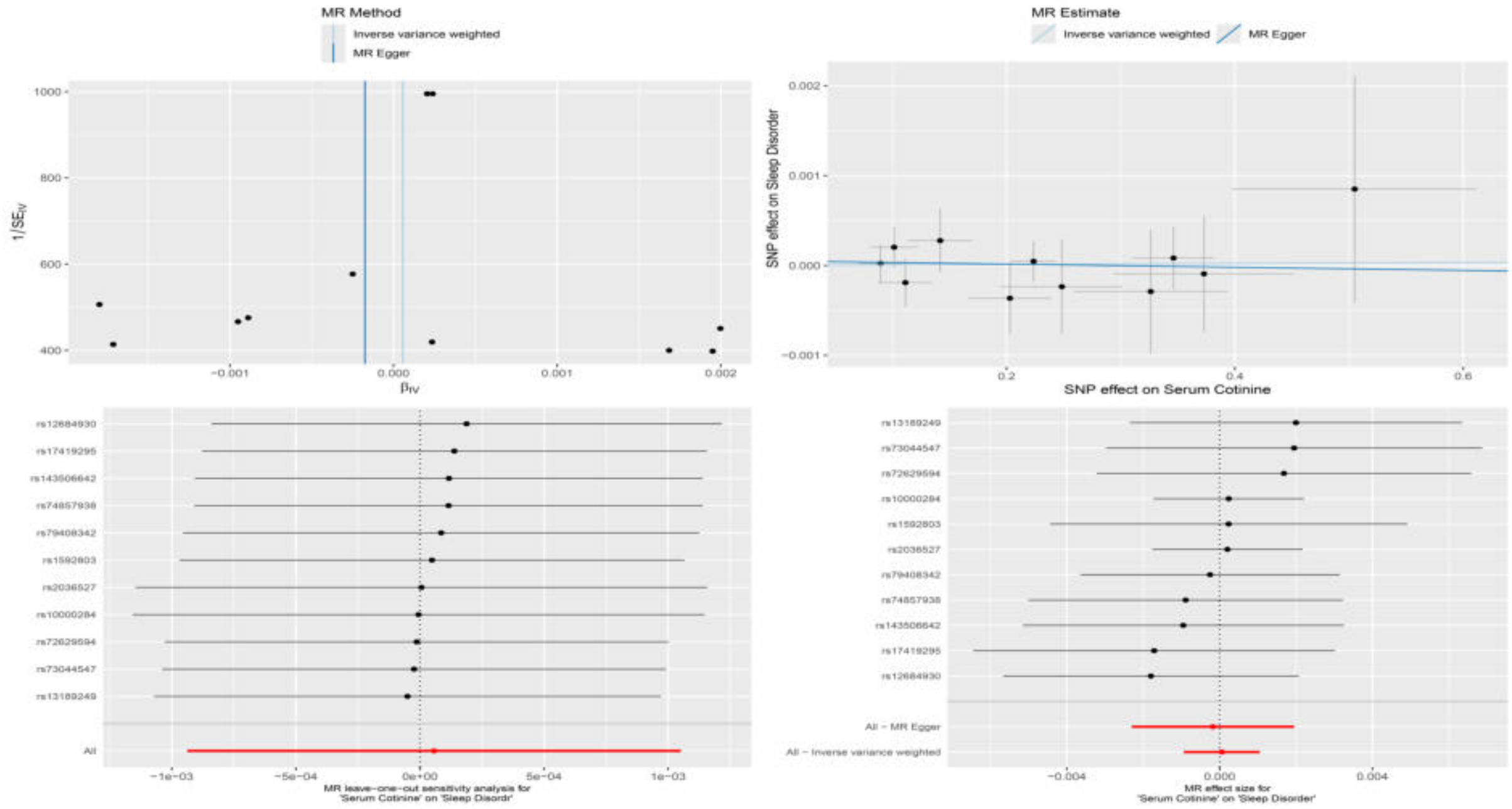

Supplement: Supplementary file 1 [file TID-24-136-s1.pdf]
